# Treatment of age-related neovascular macular degeneration: the patient’s perspective

**DOI:** 10.1007/s00417-017-3739-1

**Published:** 2017-08-04

**Authors:** S. Müller, C. Ehlken, U. Bauer-Steinhusen, W. Lechtenfeld, Z. Hasanbasic, H. Agostini, T. Wilke

**Affiliations:** 1 IPAM - Institute for Pharmacoeconomics and Medication Logistics, University of Wismar, Alter Holzhafen 19, 23966 Wismar, Germany; 2 Department of Ophthalmology, Universitäts-Klinikum Schleswig-Holstein, Campus Kiel, Germany; 3 Bayer Vital GmbH, Medical Affairs, Kaiser-Wilhelm-Allee 70, 51373 Leverkusen, Germany; 4 PRO RETINA Deutschland e.V, Vaalser Str. 108, 52074 Aachen, Germany; 5 Eye Center, Medical Center, Faculty of Medicine, University of Freiburg, Killianstraße 5, 79106 Freiburg, Germany

**Keywords:** Neovascular age-related macular degeneration, Anti-VEGF therapy, Patient perspective, Prospective non-interventional cohort study, Germany

## Abstract

**Objectives:**

The aim of this study was to assess patients’ views and expectations with regard to neovascular age-related macular degeneration (nAMD) and intravitreal anti-VEGF therapy (IVT).

**Methods:**

We conducted a multicenter, non-interventional, prospective cohort study including nAMD patients treated with IVT in Germany. Patients with at least one IVT before study enrollment and aged ≥50 years were included. Three telephone interviews were conducted during a 12-month observational period. Here, patient’s beliefs/expectations with regard to the nAMD disease and the IVT treatment were discussed. Only patients who completed all three phone interviews were included in the analyses. We used a two-step cluster analysis to identify patient clusters regarding specific patient attitudes towards nAMD and its treatment.

**Results:**

Three hundred and thirty-two patients completed all interviews (mean age of 76.4 ± 7.2 years, 59.0% women). Out of these, 57.8% acknowledged that they needed general assistance in daily life, while 77.4% stated being able to attend general medical appointments on their own. However, 64.7% needed a driver or an accompanying person to attend their IVT appointments.

In addition, 3.9% of the patients were afraid of IVT side effects. Also, 87.3% and 43.1% of the patients could name their disease or the anti-VEGF drug administered, respectively. More than three-quarters of the patients (83.1%) were aware of possible consequences of nAMD by stating vision loss or blindness, but only 16.6% knew that nAMD is a chronic disease.

Generally, patients were optimistic: 70.2%, 5.1% and 13.0% of them expected stable visual acuity (VA), a significant improvement or expected worsening of VA in the next year, respectively. Almost two thirds of patients who provided their therapy expectations (47.0%) anticipated fewer injections/discontinuation of IVT.

We identified five patient clusters differing significantly from each other with regard to four variables: being afraid of IVT, nAMD disease awareness, optimism with regard to effectiveness of IVT, and nAMD disease and treatment knowledge.

**Conclusions:**

Only a minority of patients is aware of the chronic nature of nAMD. To motivate patients to accept a life-long IVT treatment, physicians and caregivers must know that there exist different patient types with significant differences in communication needs.

## Introduction

Recent evidence shows that anti-VEGF treatment of neovascular age-related macular degeneration (nAMD) with ranibizumab and bevacizumab is not as effective in the everyday clinical setting as in clinical trials [1–7]. Observational studies with ranibizumab have shown that a treatment regimen with monthly eye examinations has not been established in everyday clinical practice [1, 6, 8–10]. Also, fewer injections are given in the everyday clinical setting than in prospective clinical studies [8]. It can be assumed that treatment-related causes, such as the logistics of arranging monthly follow-ups and regular injections, and healthcare system-related causes, such as reimbursement for injections and co-payments, may be underlying factors in this. Long-term courses of injections and regular follow-ups are not easy to adhere to, especially for the elderly [11], and therefore patient-related causes such as non-adherence or immobility may also contribute to the lower effectiveness of intravitreal treatment (IVT) in the everyday clinical setting, especially if the patients are to be followed up on a monthly basis.

Generally, every treatment should be tailored to the needs of the patient and their disease. Treatments that are effective in clinical trials, but have low patient acceptance, are likely to be less effective in everyday clinical practice because of inadequate patient adherence [12, 13]. It is obvious that this is specifically the case if, as in nAMD, life-long treatment is required. Acceptance by patients depends on several factors, such as patient treatment expectations and experiences, but also effectiveness of communication between physicians and patients [14].

The collection of reliable and valid data on nAMD patient perceptions and preferences is therefore important [15]. This is independent of the specific IVT regimen used (fixed injections, pro re nata (PRN), treat-and-extend, observe-and-plan) as all of them only work in real life if patients are willing and able to adhere to them [16].

Recent preference analyses have shown that nAMD patients are not willing to accept suboptimal visual acuity (VA) development, even if associated with a lower treatment burden [11, 15]. Little research has so far been done into general patient attitudes to nAMD, treatment expectations and fears, and the subjective evaluation of previous IVT. Specifically, nAMD patients have not been investigated to establish whether they have uniform views in this respect or whether there are distinct nAMD patient groups in need of different communication strategies.

The main purpose of this analysis in treatment-experienced nAMD patients in Germany was to assess patients’ views on having nAMD (including disease knowledge) and to evaluate intravitreal anti-VEGF therapy (IVT) from the patient perspective (including treatment expectations). We also assessed whether specific patient groups with regard to patient views on disease and treatment could be identified.

## Methods

### Setting

We conducted a multicenter, non-interventional, prospective cohort study observing nAMD patients in Germany treated with IVT. The study protocol was examined and approved by the ethics committees of the Universities of Greifswald, Rostock and Freiburg. Informed consent was obtained from all patients. Patients with nAMD were enrolled at twenty-three randomly selected treatment centers of different sizes (hospitals, office practices, outpatient clinics). Patients with at least one IVT before study inclusion (regardless of the administered agent), aged 50 years or older, and willing and able to conduct three 30-min telephone interviews in the German language were eligible. Participation in clinical studies for AMD was not allowed. To ensure that the real-life treatment of nAMD patients is observed, no further restrictions regarding the treatment course in the prospective observation period of the study were defined. Thus, patients who decided to discontinue the therapy or for whom the physician decided to stop the IVT during the following 12-months study period were also included in the sample.

### Data collection

The attending physicians documented basic sociodemographic and clinical characteristics of patients including time of first nAMD diagnosis, time of first IVT, development of VA and central retinal thickness (CRT), measured by optical coherence tomography (OCT), ophthalmological and other comorbidities as well as the data of examination and injection visits (retrospective documentation referring to the last 24 months). All eye-specific parameters were documented for the study eye (first eye treated with IVT, in case of bilateral therapy start, the eye with the worse VA was defined as study eye) and the fellow eye. Patients were expected to remain in the study for an observation period of 12 months, during which three structured telephone interviews using pre-defined questionnaires were conducted between September 2011 and October 2013 (mean prospective observation time: 344.9 days). The decision for conducting three different interviews was made to minimize the risk of overstraining of patients and consequently to ensure that the maximum duration of an interview did not extend 30 min. Furthermore, the content of the interviews was divided regarding the sensitivity of the questions; to minimize potential effects on patients’ behavior during the study period, questions with a more general nature were asked in the first interview, whereas the treatment-related and patient behavior-related questions,which might have had an influence on patients’ views or behavior, were included in the last interview. The first interview took place approximately two months after study inclusion, and consisted mainly of questions dealing with the well-being at the time of the interview and quality of life of the patient, the living environment, and the need for assistance with everyday life activities and health-related activities. The second interview five to six months after study inclusion covered topics such as the patients’ fears with regard to the disease and its treatment, and their disease knowledge and awareness. Patients were asked for a subjective assessment of vision. This was done for both eyes using a 6-point Likert scale (excellent, good, moderate, bad, very bad, blind). The third interview was conducted at the end of the observational period. It covered the patients’ beliefs, expectations, a subjective assessment of vision, and their preferences for different treatment regimens. This interview also included a discrete choice analysis of patient preferences; respective results were published separately [15]. Supplemental Table A shows all used questionnaires.

The interviews were conducted by trained interviewers who had previously conducted three to five test interviews under supervision. The data were collected in real time using an online tool developed for this purpose, which included predefined data entry fields to reduce the risk of data entry errors and missing data.

### Analyses

We included only patients in our analyses who participated in all three phone interviews. Missing data were not imputed. The patients’ responses were analyzed using descriptive statistics including means and standard deviations (SD) for continuous variables and patient counts and percentages for categorical variables. Potential differences of variables between patient groups were evaluated with the Pearson’s chi-squared test for categorical variables and with the Mann-Whitney U-test for continuous variables.

We used a two-step cluster analysis to identify patient clusters with regard to specific patient attitudes towards the nAMD disease and its treatment. This approach is the appropriate method for cluster identification if categorical data and data sets containing more than 200 subjects are available [17]. The similarity between two clusters was analyzed using the distance measure Log-likelihood. The ‘best fitted’ number of clusters was determined by using the clustering algorithm Bayesian Information Criterion. After having identified patient attitude clusters, we described these in terms of size (patient numbers), sociodemographic characteristics of patients, VA as measured in study sites, and attitudes to nAMD treatment. Differences between the clusters were analyzed using Pearson’s chi-squared test for nominal variables and the non-parametric Mann-Whitney U-test for independent samples for continuous variables. The statistical analysis was conducted using SPSS software (v. 19.0, IBM, Chicago, IL, USA).

## Results

### Study sample

Of the 480 patients enrolled with nAMD, 332 were willing and able to conduct all three phone interviews. The patients interviewed had a mean age of 76.4 ± 7.2 years at baseline and 59.0% were women (Table 1). The nAMD had been diagnosed a mean of 1.7 ± 1.9 years (range 0.0–9.3 years) before study inclusion, and the first IVT had been given a mean of 1.4 ± 1.5 years (range: 0.0–6.4 years) before study inclusion. Before study inclusion, the patients received on average 5.3 ± 3.8 intravitreal injections (based on a 24-months retrospective documentation period). During the observational period, 73.2% of the patients were treated with ranibizumab, 51.2% with bevacizumab, 4.5% with aflibercept and 1.2% received pegaptanib (double-counting possible). The mean VA of the study/fellow eye was 0.6 ± 0.5 logMAR/0.7 ± 0.6 logMAR at baseline, and the median VA was 0.5 logMAR/0.5 logMAR.

**Table 1:** Sociodemographic characteristics of patient samples at the time of study inclusion

Variables collected at the time of inclusion	All study patients	Study patients, who took part in all three phone interviews during the observation period (study sample)	Study patients who did not take part in all three phone interviews
N	480	332	148
Age in years*	77.27 (range 53–94; SD 7.4)	76.39 (range 53–92; SD 7.2)	79.25 (range 55–94; SD 7.5)
Women	289 (60.2%)	196 (59.0%)	93 (62.8%)
Time in years since first nAMD diagnosis	1.75 (range 0.02–9.31; SD 1.9)	1.68 (range 0.02–9.31; SD 1.9)	1.89 (range 0.02–8.08; SD 2.0)
Time in years since first IVT	1.42 (range 0.01–6.36; SD 1.5)	1.39 (range 0.01–6.36; SD 1.5)	1.49 (range 0.01–5.98; SD 1.6)
Mean number of IVIs administered before study inclusion (24-months retrospective documentation period)	5.23 (range 0–20; SD: 3.8)	5.32 (range 0–20; SD: 3.8)	5.10 (range 0–20; SD: 3.9)
Fellow eye with nAMD	216 (45.0%)	146 (44.0%)	70 (47.3%)
Mean and median visual acuity of study eye in logMAR*	Mean: 0.68 (range 0.05–2.3; SD 0.5) Median: 0.6	Mean: 0.64 (range 0.05–2.3; SD 0.5) Median: 0.5	Mean: 0.76 (range 0.05–2.3; SD 0.5) Median: 0.5
Mean and median visual acuity of fellow eye in logMAR	Mean: 0.71 (range − 0.10-2.0; SD 0.6) Median: 0.5	Mean: 0.71 (range 0.00–1.7; SD 0.6) Median: 0.5	Mean: 0.73 (range − 0.10-2.0; SD 0.5) Median: 0.6

^a^Differences were evaluated for statistically significant differences between the group of patients excluded due to incomplete interviews and the group with completed interviews. We used Pearson’s Chi-squared-test for nominal variables and the Mann-Whitney U-test for continuous variables; *a significant difference was seen for age (*p* < 0.001) and visual acuity of the study eye at time of inclusion (*p* < 0.050)

SD: standard deviation; IVT: intravitreal therapy; IVI: intravitreal injection; logMAR: logarithm of the minimum angle of resolution; nAMD: neovascular age-related macular degeneration

The 148 patients who were not willing or able to conduct phone interviews were statistically significantly older than the 332 patients included in this analysis (mean of 3 years; *p* < 0.001) and had a worse mean VA of the study eye (0.76 logMAR versus 0.64 logMAR; *p* < 0.050) (Table 1).

### Home environment and patient well-being

About two-thirds of the patients lived together with their spouse (62.3%), and about one third lived alone (28.0%). In addition, 8.1% of the patients lived together with their children or other relatives, and 1.5% lived in care homes or an assisted living environment.

**Fig. 1:**
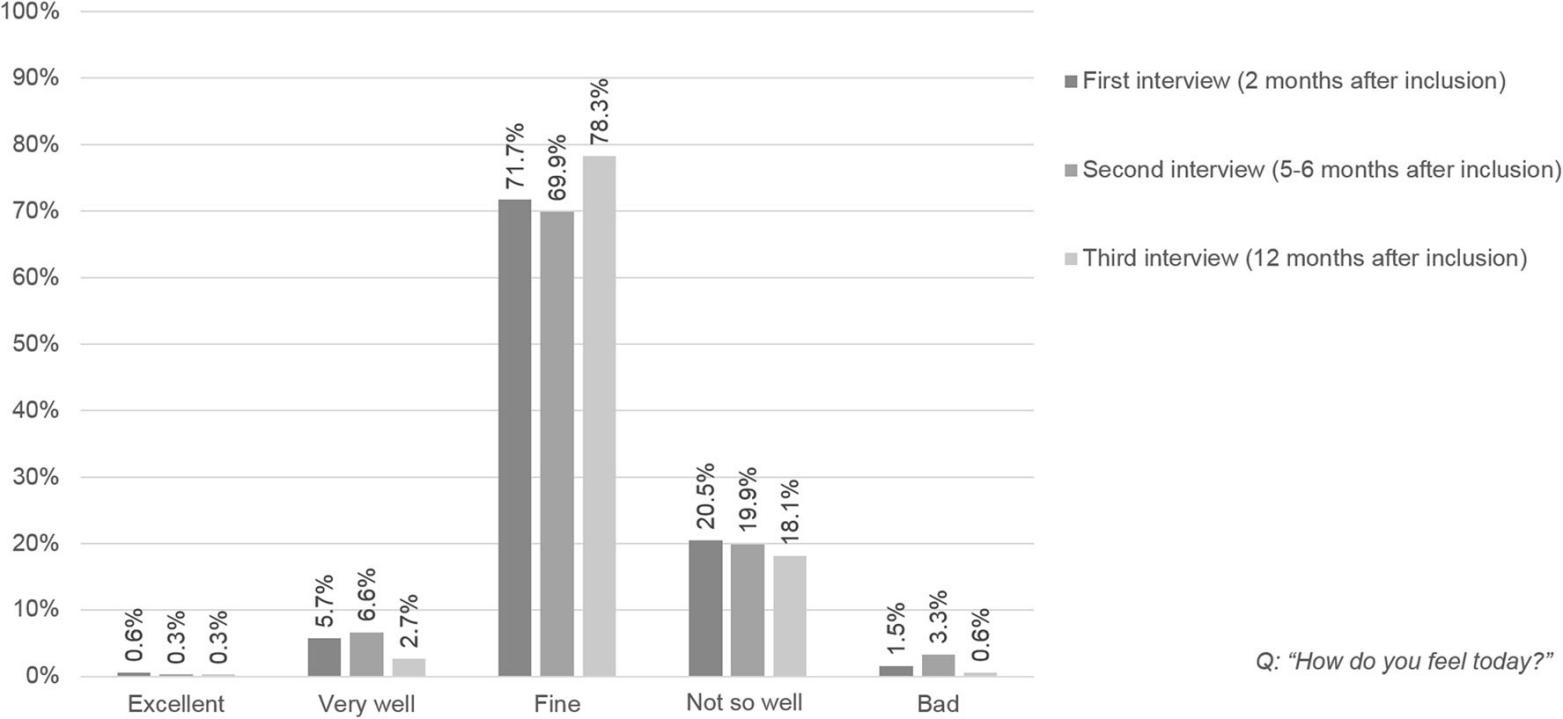
Subjective well-being at the time of the interviews (*n* = 332)

**Fig. 2:**
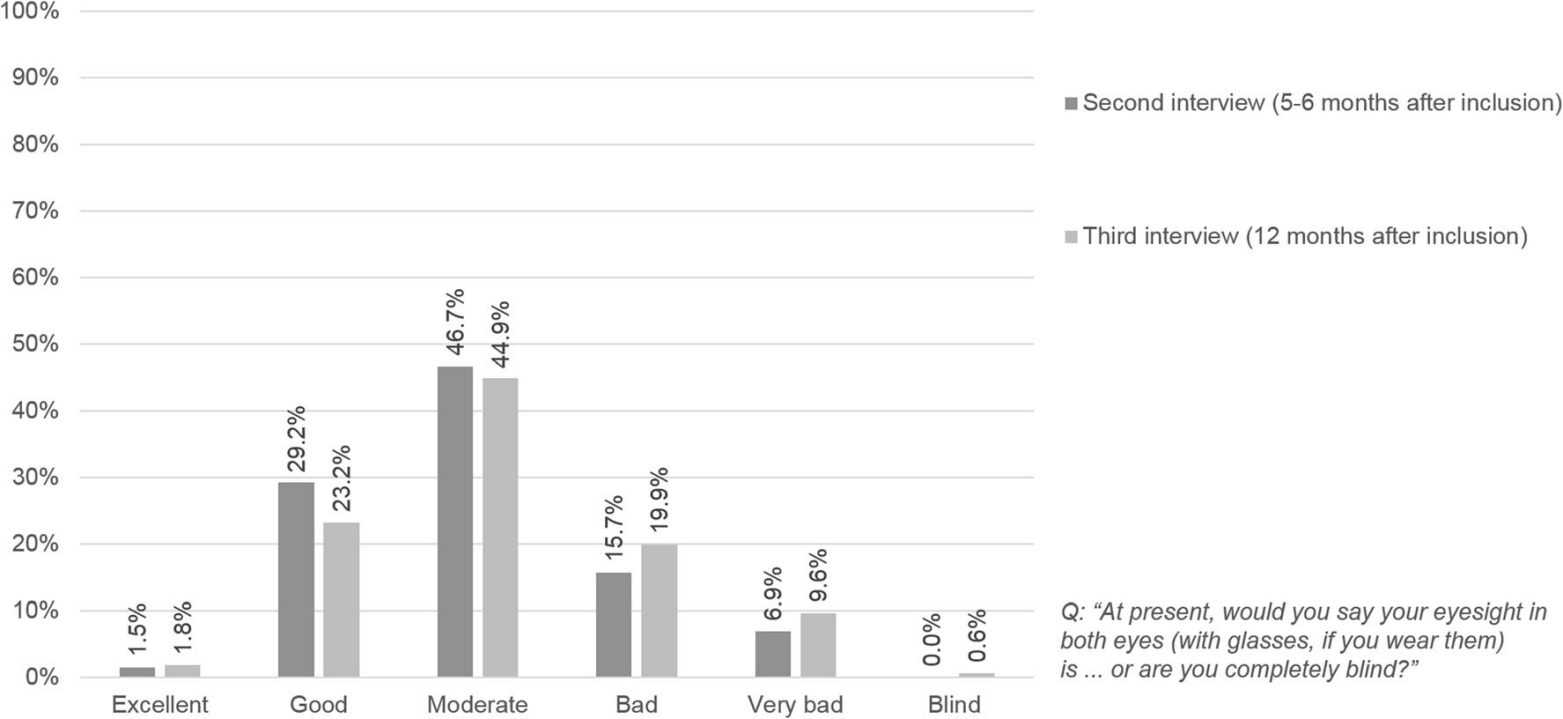
Subjective assessment of change in vision between second and third interview (*n* = 332)

The majority of patients (first interview: 71.7%; second interview: 69.9%; third interview: 78.3%) described their well-being at the respective interview as moderate, and this was relatively stable over the 12-month observation period (Fig. 1). Only a few patients (6.6% and 2.7% of patients across interviews) stated that they felt very well, and approximately one fifth stated feeling less well. The subjective assessment of vision did not change between the second and the third interview (interval of about six months) in 31.9% of the patients. In 32.2% of the patients, the subjective assessment of vision deteriorated, and in 19.6% of the patients it improved. The percentage of patients who assessed their vision as at least good decreased from 30.7% to 25.0%, and the proportion that assessed it as bad or very bad increased from 22.6% to 29.5% (Fig. 2).

### Independence and need for assistance

**Fig. 3:**
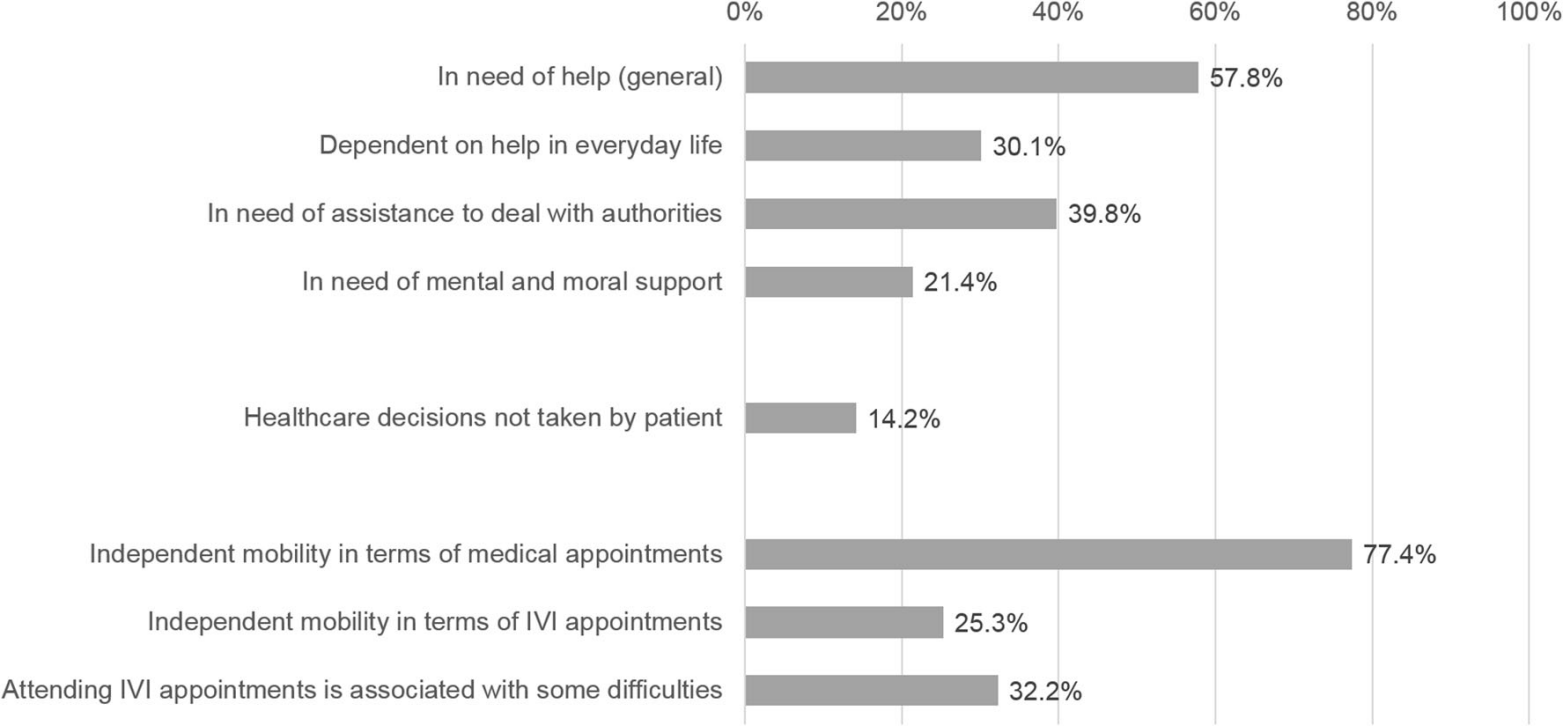
Independence and need for assistance in interviewed patients assessed by data collected in the first interview (*n* = 332)

Overall, 57.8% of the 332 patients stated that they are generally in need of help (Fig. 3). Of these, 52.1% needed assistance in everyday life, 68.8% stated they needed help in dealing with authorities, and 37.0% needed mental and moral support.

In 14.2% of the patients, healthcare decisions were made by a third party. About three quarters of patients stated that they were able to attend general medical appointments on their own. In contrast, about two thirds needed a driver or an accompanying person to attend their appointments for IVT, as car driving is not recommended for patients after treatment. In most cases, a relative accompanied the patient. Also, 32.2% of patients reported that attending IVT appointments were associated with difficulties (Fig. 3).

### Fear of IVT, nAMD disease and treatment knowledge, and disease awareness

Moreover, 9.6% of the patients professed to be afraid of injections in general. Only 3.9% were afraid of the side effects of IVT. Nevertheless, 66.3% of the patients would prefer tablets or eye drops instead of intravitreal injections (IVIs), if equally effective. The percentage of patients with only one IVT in the time before study inclusion was slightly higher in the group of patients who stated to be not afraid (14.7% vs. 10.3%); this difference did not reach statistical significance (*p* = 0.626).

**Fig. 4:**
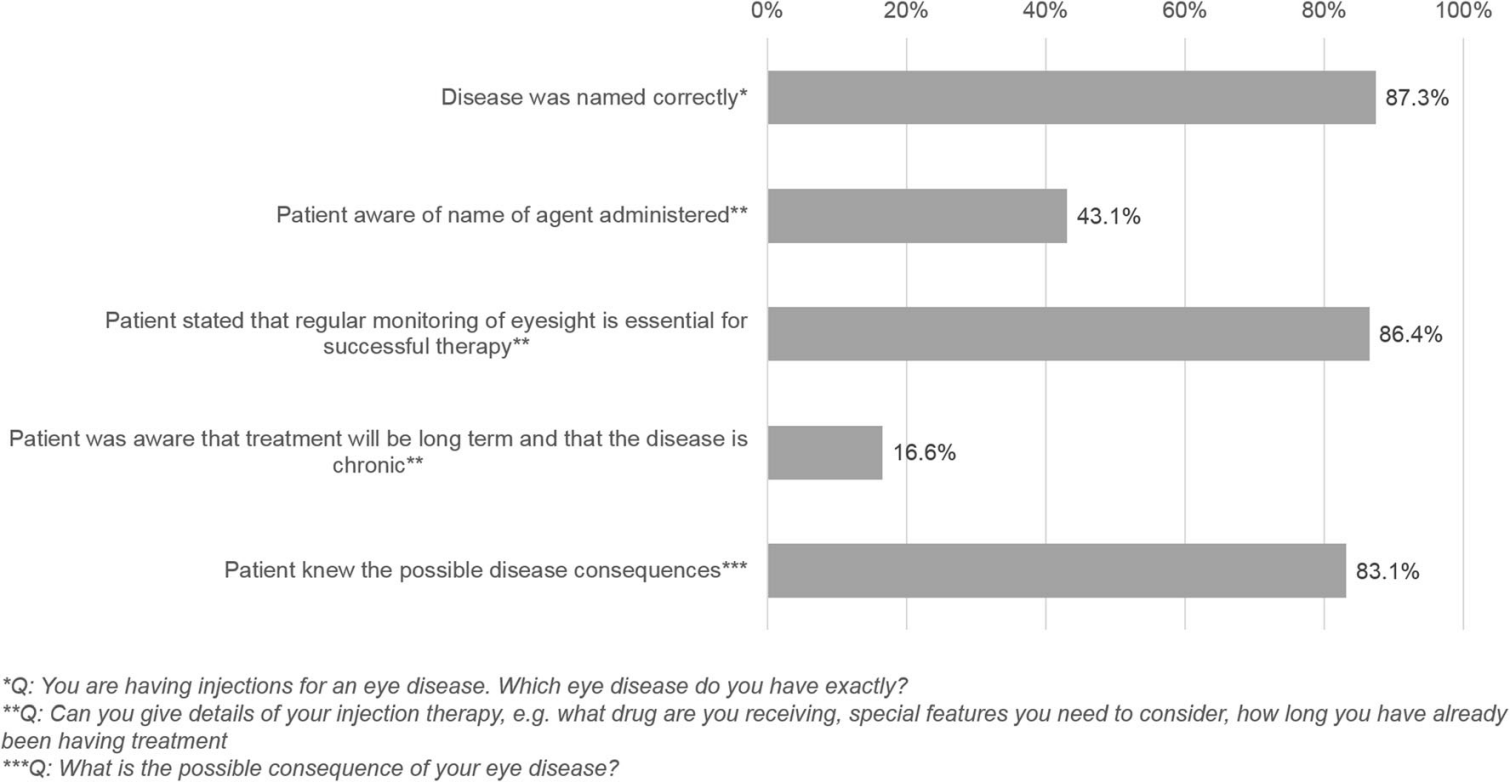
Patients’ knowledge about nAMD disease and treatment assessed by data collected in the second interview (*n* = 332)

Several disease- and treatment-specific questions assessed the patients’ knowledge of their disease and treatment (Fig. 4). Furthermore, 87.3% of the patients named their eye disease correctly, and 43.1% were able to name the anti-VEGF drug administered. Then 31.1% reported paying particular attention to their diet because of their eye disease (e.g., eating broccoli, cabbage, carrots), and 30.4% were taking special vitamin supplements.

More than three quarters were aware that nAMD may result in vision loss or blindness (83.1%), and a similar proportion (86.4%) was aware that regular monitoring of VA is essential for successful therapy, and 61.2% of patients reported using self-tests for the subjective assessment of their VA. However, only 16.6% of the patients were aware of the chronic nature of the nAMD disease, and most of the patients hoped or believed that the IVT was necessary only temporarily.

### Patients’ beliefs and expectations

The 332 patients were asked how their treatment expectations had changed since the start of IVT. About half (53.0%) professed to be as optimistic as at the beginning of therapy; 15.4% stated that they were more optimistic than at the time of the first anti-VEGF injection (Table 2). Only 13.6% stated that they were less optimistic than at the beginning. In line with this, 86.1% of the patients stated that they would start therapy again.

**Table 2:** Overview of patient expectations collected within the third phone interview at the end of the study (*n* = 332)

Categories	Attributes	n (%)
Change in attitude or expectations towards treatment since first injection	As optimistic as at the beginning of the therapy	176 (53.0%)
	More optimistic than at the beginning of the therapy	51 (15.4%)
	As pessimistic as at the beginning of the therapy	17 (5.1%)
	More pessimistic than at the beginning of the therapy	45 (13.6%)
	Not able to answer the question	43 (13.0%)
	Number of patients who would start therapy again	286 (86.1%)
Expectations regarding injection therapy in the next year	Expected significantly more injections	58 (17.5%)
	Expected significantly fewer injections	40 (12.0%)
	Expected no further need for injections (‘cure’)	14 (4.2%)
	Expected to discontinue treatment (self)	15 (4.5%)
	Expected discontinuation of treatment (by physician)	29 (8.7%)
	Not able to answer the question	176 (53.0%)
Expectations regarding the development of visual acuity in the next year	Expected a significant improvement in visual acuity	17 (5.1%)
	Expected visual acuity to remain the same	233 (70.2%)
	Expected a significant worsening in visual acuity	43 (13.0%)
	Expected blindness	2 (0.6%)
	Not able to answer the question	37 (11.1%)

The patients’ general optimism was also reflected in their expectations with regard to the development of VA: 70.2% expected that their VA would remain the same during the next year, and 5.1% expected a significant improvement. Also, 13.0% believed that their VA would significantly worsen, and two patients expected blindness.

About half of the patients (53.0%) were not able to express any expectations regarding future injection therapy; the most frequent reason (39.8%) was that the attending physician decided on the course of therapy, and; thus, the patient did not have any treatment-related expectations.

Based on the patients who stated expectations at the time of the interview, 37.2% expected significantly more injections and 25.6% expected significantly fewer injections in the next 12 months. In addition, 9.0% believed that the therapy would cure them in the next year so that no more injections would be needed, 9.6% expected to discontinue therapy in the next 12 months; and 18.6% expected that the physician would decide to discontinue the therapy in the next 12 months (Table 2).

### Patient clusters identified

Based on our statistical analysis, we were able to identify the following predictors as dichotomous independent parameters for assignment of patients to a specific cluster:

- Presence of fear: patients who stated that they were afraid of injections or side effects, or both, versus those who were not afraid.
- Awareness: patients who applied regular VA self-tests and paid attention to their diet, including taking vitamin supplements, because of their eye disease, versus those who did not apply self-tests and did not care about their diet.
- Optimism with regard to treatment: patients who stated that they were more optimistic or at least as optimistic as at the time of treatment initiation, versus those who professed to be pessimistic.
- Knowledge of disease and treatment: patients who correctly answered at least four out of the five questions about nAMD disease and treatment, as shown in Fig. 4.

Considering these predictors, five different patient clusters were identified (Table 3). The clusters identified showed an average silhouette of cohesion and separation of 0.7 and thus a good cluster quality. The first cluster contained 94 (28.3%) optimistic patients with good disease awareness, without fear of injections or side effects, and a below average knowledge of their disease and treatment knowledge. The second cluster contained 74 (22.3%) patients who differed from the patients in the first cluster only with regard to their better knowledge of their disease and treatment. The 65 patients (19.6%) allocated to the third cluster were patients not afraid of injections or side effects, had good disease awareness and an average knowledge of their disease and treatment, but were pessimistic with regard to their expectations of therapy. Assignment to the fourth cluster was mainly driven by the predictor ‘(non)-awareness’ of disease and comprised 60 patients (18.1%) who did not use self-tests and did not pay attention to their diet. The fifth cluster contained the 39 patients (11.7%) afraid of injections or side effects or both. Table 3 shows the age and gender as well as main treatment-related characteristics of the patients in the different clusters.

**Table 3:**
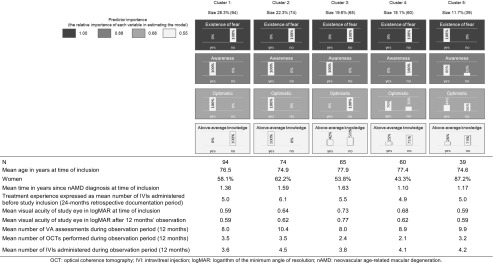
Description of patient segments identified, including patient characteristics (*n* = 332)

OCT: optical coherence tomography; IVI: intravitreal injection; logMAR: logarithm of the minimum angle of resolution; nAMD: neovascular age-related macular degeneration

Patients in the first cluster showed both a better mean VA at the beginning and at end of the observation period than all other patients. The difference was, however, not statistically significant. Furthermore, optical coherence tomography was applied more frequently in these patients (3.5 versus 2.8, *p* = 0.045), while VA measures were less frequently conducted (8.0 versus 9.3, *p* = 0.009). Patients in the second cluster who had – in contrast to the other patients – an above-average knowledge of their disease and treatment were significantly younger (74.9 versus 76.8 years, *p* = 0.016) and had a longer disease history (time from first diagnosis to study inclusion 1.59 versus 1.6 years, *p* = 0.047). In these patients, significantly more VA assessments were applied during the 12-month observation period (10.4 versus 8.5, *p* = 0.002). The pessimistic patients (cluster 3) differed from all the other patients mainly with regard to VA. Their VA was worse at the beginning (0.73 versus 0.62 logMAR, *p* = 0.015) and end of the observation period (0.77 versus 0.60 logMAR, *p* = 0.009). The fourth cluster, which consisted of patients with a lack of awareness, comprised significantly more male patients (56.7% versus 37.6%, *p* = 0.007). In this group, fewer optical coherence tomography investigations were made during the 12-month observation period (2.1 versus 3.2, *p* = 0.006). The last cluster with patients afraid of injections and side effects contained significantly more women than men (87.2% versus 55.1%, *p* < 0.001) as compared to the other clusters.

## Discussion

The main purpose of this paper, based on a survey of IVT-experienced nAMD patients in Germany, was to assess their views on nAMD disease, including disease knowledge, and to evaluate an IVT treatment from the patient perspective. We also investigated whether specific patient groups could be identified with regard to their views on and attitudes towards disease and treatment.

In line with existing publications [18], we observed that the majority of our treatment-experienced nAMD patients are in need of some form of assistance. About two thirds of our patients needed a driver or an accompanying person to attend their appointments for IVT – in most cases, a relative. Despite existing treatment experience, about 10% stated that they were afraid of IVT. About nine out of ten patients confirmed that regular monitoring of VA is essential for successful therapy, and six in ten patients reported using self-tests (mostly the Amsler grid) for the subjective assessment of their vision. This generally high acceptance rate was met by a high degree of optimism with regard to IVT, such that about nine out of ten nAMD patients would start the IVT again. A cross-sectional survey in a hospital eye clinic in France (including 58 patients lost to follow-up) showed that patients’ acceptance is an important predictor of therapy continuation and identified, that in approximately one-fourth of the patients, who discontinued the therapy, the excessive burden of periodic follow-up visits and, in nearly 35% of the patients a subjective dissatisfaction with the benefits of intravitreal injections, led to therapy discontinuation [8].

It is interesting to note that, whereas about eight out of ten patients were aware of the possible consequence of nAMD disease, namely vision loss or blindness, only two out of ten patients were aware of the chronic nature of the disease. In addition to this, only one out of ten patients believed that the VA would significantly worsen in future, and about six out of ten expected either significantly fewer injections or discontinuation of IVT in the next 12 months. Contrary to this existing lack of patient knowledge, a study in Switzerland conducting phone interviews with AMD patients showed that the patients seem to be willing to get more information about their specific disease situation [19].

We identified different patient clusters based on their responses to our questionnaires. To our knowledge, no scientific analysis has so far attempted to identify differences between nAMD patients’ opinions and expectations, and to form different patient clusters in this respect. Comparison of our segmentation results with the literature is therefore not possible.

A review from 2012 analyzing qualitative studies related to the perspective and needs of AMD patients concluded, that a holistic approach to service provision and support for AMD is needed which considers individuals’ needs and experiences when coping with AMD [20]. In this context, we believe that our results are important for the development of future nAMD patient communication strategies for the following reasons: (1) Patient acceptance of IVT and patient understanding and acceptance of the long-term nature of the disease and therapy are necessary to maintain long-term IVT. As a result of our investigation, obviously, the majority of patients believe that nAMD is a temporary disease, which can be either cured by an IVT or progression can be stopped by such a therapy. (2) Patients differ in terms of treatment optimism, disease awareness and knowledge, and fear of injections or side effects. To achieve the high degree of patient acceptance required, we recommend the development of separate patient communication strategies for the five patient clusters we identified. For example, improving nAMD knowledge should be the focus of communication in cluster 1 patients, whereas communication with cluster 3 patients should target the generally pessimistic view of IVT.

We acknowledge some limitations of our study. First, despite random selection of study centers with consecutive patient inclusion, there may still have been selection bias on the part of the physicians involved. Second, we were able to conduct all three telephone interviews in only 332 out of 480 patients resulting in a possible response bias. A further limitation is that we included only IVT-experienced nAMD patients. At the time of the interviews, patients were almost exclusively being treated with ranibizumab or bevacizumab (96%), mostly PRN (73%). This might have biased the patients’ opinions expressed in the interviews. The patients’ opinions could also be influenced by the different length of intervals between the last or the next IVT and the interview or by the treatment experience of the patient. However, no significant differences in the duration of treatment intervals or number of previous administrated intravitreal injections were found within the different patient groups. Nevertheless, it needs to be acknowledged that we included a general sample of treatment-experienced patients who could have recently started their IVT therapy, but could have also started their therapy before our (retrospective 24 month) observation began.Therefore, we could not access whether previous treatment experience influenced patients’ opinions, as patients’ views were not assessed from the start of the therapy onwards. Furthermore, we did not investigate recently introduced treatment regimens such as the treat-and-extend strategy, which is widely used, particularly in the USA [21], or the observe-and-plan scheme. We also acknowledge that we were unable to assess whether the poor VA and a less-effective treatment in patient cluster 3 led to a pessimistic view of the IVT or whether the pessimistic view itself was a contributory factor in the subsequent poor treatment results.

### Conclusions and implications for practice

Only a minority of nAMD patients are aware of the chronic nature of their disease. To motivate nAMD patients in accepting the necessary long-term treatment, physicians and caregivers must be aware of the differences between the degrees of disease knowledge and the opinions and expectations of nAMD patients. The communication strategy should be suited to the individual patient. Adapted communication strategies should be investigated to establish whether they result in enhanced patient persistence.
